# Supplementary material for: Synbiotic Intervention with an Adlay-Based Prebiotic and Probiotics Improved Diet-Induced Metabolic Disturbance in Mice by Modulation of the Gut Microbiota
Source: Nutrients. 2021 Sep 10;13(9):3161. doi: 10.3390/nu13093161 (PMC8471612; doi:10.3390/nu13093161)
Supplement: Supplementary file 1 [file nutrients-13-03161-s001.zip › nutrients-1365926-supplementary.pdf]

Table S1. Primers.

Primer name	Forward primer (5'-end to 3'-end)	Reverse primer (5'-end to 3'-end)
MCP-1	TTAAAAACCTGGATCGGAACCAA	GCATTAGCTTCAGATTTACGGGT
TNF-a	TGGGAGTAGACAAGGTACAACCC	CATCTTCTCAAAATTCGAGTGACAA
IL-6	CCAGTTGCCTTCTTGGGACT	GGTCTGTTGGGAGTGGTATCC
LBP	GTGGCTGCTGAATCTCTTCC	GAGCGGTGATTCCGATTAAA
Leptin	TCTCCGAGACCTCCTCCATCT	TTCCAGGACGCCATCCAG
CD11c	CTGGATAGCCTTTCTTCTGCTG	GCACACTGTGTCCGAACTC
GAPDH	AACTTTGGCATTGTGGAAGG	GGATGCAGGGATGATGTTCT
